# Nanostructured Thin Films Obtained from Fischer Aminocarbene Complexes

**DOI:** 10.3390/ma9030167

**Published:** 2016-03-04

**Authors:** Rosa E. Lazo-Jiménez, M. Carmen Ortega-Alfaro, José G. López-Cortés, Cecilio Alvarez-Toledano, José Á. Chávez-Carvayar, Jordi Ignés-Mullol, Maykel González-Torres, Pilar Carreón-Castro

**Affiliations:** 1 Instituto de Ciencias Nucleares, Universidad Nacional Autónoma de México, A. Postal 70-543 Circuito Exterior, Ciudad Universitaria, México, D.F. 04510, Mexico; estela.lazo@nucleares.unam.mx (R.E.L.-J.); carmen.ortega@nucleares.unam.mx (M.C.O.-A.); 2 Instituto de Química, Universidad Nacional Autónoma de México, Circuito Exterior, Ciudad Universitaria, México, D.F. 04510, Mexico; jglcdw@unam.mx (J.G.L.-C.); cecilio@unam.mx (C.A.-T.); 3 Instituto de Investigaciones en Materiales, Universidad Nacional Autónoma de México (UNAM), Circuito Exterior, Ciudad. Universitaria, México, D.F. 04510, Mexico; josech@unam.mx; 4 Departament de Química-Física, Universitat de Barcelona, Martí i Franquès 1, Barcelona E-08028, Spain; jignes@ub.edu; 5 Centro de Física y Tecnología Avanzada, Universidad Nacional Autónoma de México, Querétaro 76230, Mexico; mikegcu@gmail.com

**Keywords:** LB films, ferrocene derivatives, carbene complexes, nanostructures, thin films

## Abstract

The synthesis of four amphiphilic organometallic complexes with the general formula RC = M(CO)_5_NH(CH_2_)_15_CH_3_, where R is a ferrocenyl **2**(**a-b**) or a phenyl **4**(**a-b**) group as a donor moiety and a Fischer carbene of chromium (0) or tungsten (0) as an acceptor group, are reported. These four push-pull systems formed Langmuir (L) monolayers at the air-water interface, which were characterized by isotherms of surface pressure *versus* molecular area and compression/expansion cycles (hysteresis curves); Brewster angle microscopic images were also obtained. By using the Langmuir–Blodgett (LB) method, molecular monolayers were transferred onto glass substrates forming Z-type multilayers. LB films were characterized through ultraviolet-visible spectroscopy, atomic force microscopy and X-ray diffraction techniques. Results indicated that films obtained from **2b** complex [(Ferrocenyl)(hexadecylamine)methylidene] pentacarbonyl tungsten (0) are the most stable and homogeneous; due to their properties, these materials may be incorporated into organic electronic devices.

## 1. Introduction

During the last decade, considerable efforts have been carried out to obtain materials for electronic and optoelectronic applications, which led to molecular electronics, a new, important, and interdisciplinary field of research [1]. Although most research has been devoted to inorganic materials, few studies about organic and organometallic derivatives may also be found. In this sense, organometallic entities have attracted considerable attention due to their plausible electron transfer processes [2]. Particularly, those containing ferrocene (Fc) have emerged as an important π-conjugated donor group in the construction of “push-pull” systems, which possess charge-transfer excited states [3,4,5]. The main characteristics of these ferrocenyl derivatives can be listed as follows: quasi-reversible oxidation, chemical stability, high stability under light irradiation, and reductive quenching of excited molecules [6], being implicated in electrochemical and redox systems [7,8], luminescent, and photochromic systems [9], conductive devices [10], biosensors [11], and chemosensors [12], among others. A promising scaffold to evaluate electronic transport through conjugated frameworks are the Fischer carbene complexes, which include studies on electron transfer processes involving electronic interactions between electron-donor (D) and electron-acceptor (A) groups [13,14,15,16,17,18].

In a previous work, ferrocenyl Fischer carbenes were incorporated in thin films by thermal evaporation processes displaying a semiconductor character. In that study we found that the thin morphology strongly depended on the metallic ion in the molecular structure; thus, the materials were amorphous or poorly crystalline and, unfortunately, not all the compounds sublimate easily [19]. In order to analyze and improve the properties of these materials, in this work we report the incorporation of Fischer aminocarbenes into nanostructured LB films [20,21]. The title aminocarbenes were designed as push-pull systems, in which the ferrocenyl or phenyl fragments can act as electron-donor groups, and the corresponding Fischer carbene (Cr or W) moieties can function as strong electron-acceptor groups. The introduction of a long alkyl chain would allow the amphiphilic character necessary to make them suitable to the LB technique [22,23]. Related studies in which ferrocenyl compounds were used as precursors of LB films have found different applications in the area of materials with magnetic [24] and redox [25] properties.

The electrophilic character of the carbenic carbon in Fischer complexes facilitates the attack by nucleophiles such as primary amines. As a consequence of the electrophilic character on carbenic carbon, *α*-anions were easily generated upon treatment of alkyl carbene complexes with bases [26,27,28]. Applying this efficient strategy, we synthesized four Fischer aminocarbenes’ amphiphilic character under a push-pull architecture. The preparation of L monolayers and non-centrosymmetric *Z*-type LB multilayers and the subsequent mono- and multilayer LB-deposition onto glass substrates were also reported. The LB films were characterized by ultraviolet-visible (UV-VIS) spectroscopy, atomic force microscopy (AFM), and X-ray diffraction (XRD). The stability and morphology of these films were studied through combined isotherms, *i.e.*, surface pressure (π) *versus* molecular area (*A*), compression–expansion cycles (hysteresis), and Brewster angle microscopy (BAM). The monolayers based on ferrocenyl aminocarbene exhibited more stability in hysteresis curves than the monolayers of phenyl aminocarbene derivatives.

## 2. Results and Discussion

### 2.1. Synthesis of Organometallic Precursors

**Scheme 1:**
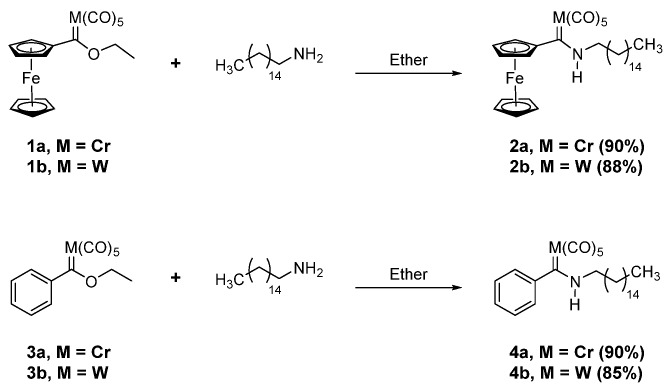
Synthesis of Fischer aminocarbenes of ferrocenyl **2**(**a-b**) and phenyl **4**(**a-b**) complexes.

The ferrocenyl (**2a-b**) and phenyl (**4a-b**) hexadecylamino carbene Fischer complexes were synthesized in good yields through an aminolysis reaction (with hexadecylamine) of the corresponding ethoxy ferrocenyl (**1a-b**) and ethoxy phenyl (**3a-b**) Fischer carbene complexes, respectively [29] (Scheme 1).

The obtaining of Fischer aminocarbenes **2**(**a-b**) and **4**(**a-b**) were confirmed by solution NMR spectra (^1^H and ^13^C), which showed the characteristic signals for these complexes. Particularly, for complexes **2**(**a-b**), we observe the only presence of *Z*-isomers. This behavior is presumably due to the bulkiness of the Fc residue, which has a steric repulsion with the side-chain, as observed in other ferrocenyl amino carbenes analogues [30]. In the case of **4**(**a-b**) complexes, they exist as mixtures of *Z*- and *E*-isomers with regard to the partial C-N double bound, being the isomer *E* the main specie, in accordance with the literature [31].

In the ^13^C-NMR spectra, the signal for carbenic carbon appeared around δ = 270 ppm (**2a**) and 281 ppm (**4a**) for chromium complexes. For tungsten series, these signals are shifted at 249 ppm (**2b**) and 258 (**4b**) ppm. In the region between δ = 223–199 ppm, we can observe the signals due to M–CO. Through infrared spectra, the four complexes displayed the typical M–CO bands around 2000 cm^−1^ and a fine band around 3300 cm^−1^ assigned to the N–H group. The observed peaks from mass spectrometry were in accordance with the expected molecular ions [(M^+^)] for all the complexes. 

**Figure 1:**
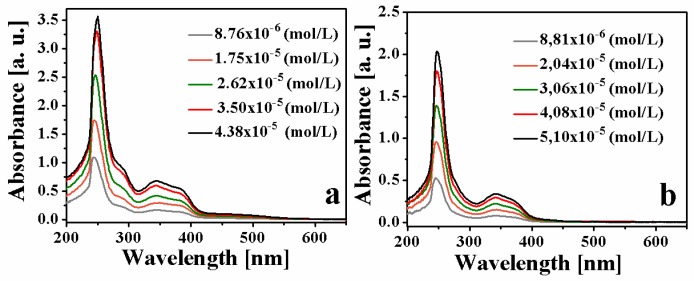
UV–VIS spectra of: (**a**) **2b** and (**b**) **4b** aminocarbene complexes in CHCl_3_ solution.

The UV-VIS spectra of **2b** showed three well-defined absorptions bands (which depend on the nature of the substituent attached to the carbene carbon atom). These bands have been identified as a spin-forbidden metal-ligand charge transfer (MLCT) band in the range between 450 and 500 nm, a moderately intense ligand field (LF) band at 387 nm, and an additional, less-intense LF band at 347 nm [3,15,32]. Compared with similar ferrocenyl derivatives, the observed bands in **2b** appeared at shorter wavelengths [3,32]. In a similar manner, the Fischer aminocarbene complex **4b** displayed three bands at 245 nm, 343 nm, and 370 nm. From this results, it is evident that the MLCT band of ferrocene derivative **2b** is slightly red-shifted compared to the phenyl derivative **4b** (Δλ = 17 nm). These results confirmed the π-donor character of the ferrocene fragment [3,15,33]. In both cases, the presence of the amino group [NH(CH_2_)_15_CH_3_] provoked a blue-shifted absorption of the MLCT and LF transitions, similarly to other Fischer-type carbene complexes [34,35]. A comparison of electronic absorption spectra for **2b** and **4b** complexes is shown in Figure 1. The data revealed that the MLCT and LF bands have a remarkable π–π* character.

### 2.2. Langmuir Monolayer Surface

The Fischer aminocarbene complexes, **2**(**a-b**) and **4**(**a-b**), were able to form L monolayers at the air-water interface. These monolayers were prepared and characterized by measuring the isotherm of surface pressure *versus* molecular area (π/*A*) and BAM observations. The L monolayers of **2b** and **4b** were also characterized by hysteresis plots.

#### 2.2.1. Langmuir Monolayers of **2a** and **4a** Complexes, with Chromium as Transition Metal

##### Langmuir Isotherm for the **2a** Complex

**Figure 2:**
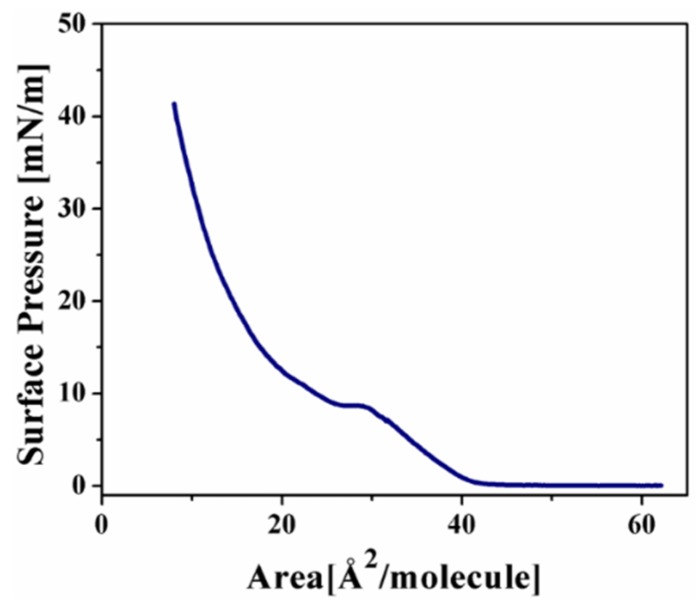
Surface pressure *vs.* area isotherm of ferrocenyl aminocarbene complex **2a**, M = Cr.

Langmuir films for the ferrocenyl aminocarbene **2a** complex (M = Cr) were prepared; the isotherm, which was recorded at 25 °C, is shown in Figure 2. A phase transition, at a surface pressure around 7 mN·m^−1^ with a molecular area 32.0 ± 2 Å^2^, was observed—*in situ*—by BAM images. Additionally, this plot shows that collapse begins with the formation of a short plateau at a surface pressure around 9 mN·m^−1^.

**Figure 3:**
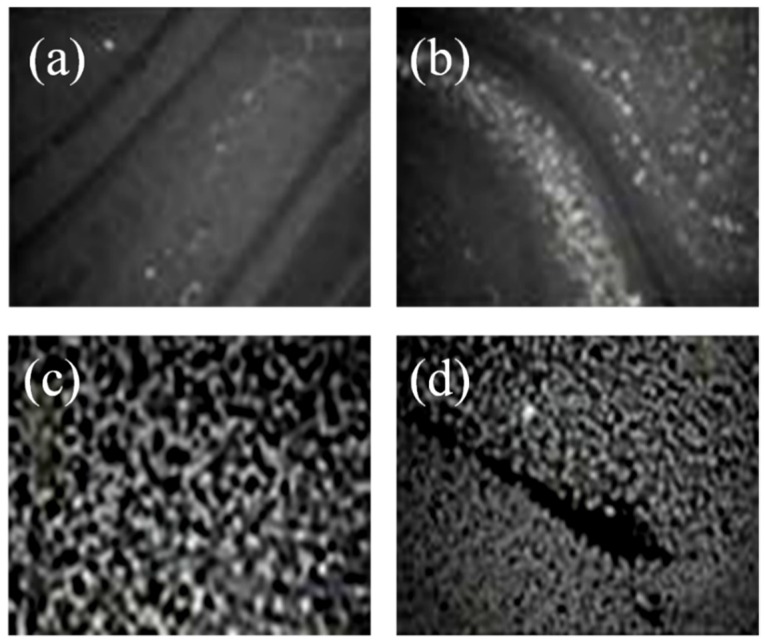
BAM images (width: 500 µm) of the **2a** compound at different surface pressures: (**a**) 0.5 mN·m^−1^, (**b**) 3.0 mN·m^−1^, (**c**) 7.0 mN·m^−1^, and (**d**) 35.0 mN·m^−1^.

BAM images, which are shown in Figure 3, confirm the formation of L film of the **2a** compound. When the surface pressure increases, bands with molecules following the water waves on the surface were observed (see Figure 3a,b). It can be observed the formation of a film with small holes that contain water producing an irregular monolayer, Figure 3c. At a higher surface pressure of 35.0 mN·m^−1^, a fold of the film was observed, Figure 3d. Some isolated large folds, which appeared between the air-water interface, may be reversible to form a monolayer when the film is expanded [36,37]. 

##### Langmuir Isotherm for the **4a** Complex

**Figure 4:**
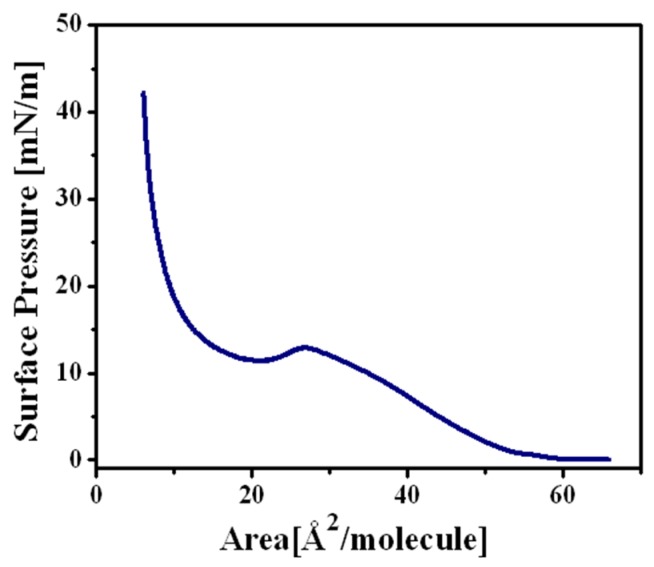
Surface pressure *vs.* area isotherm of phenyl aminocarbene complex **4a**, M = Cr.

The isotherm for the phenyl aminocarbene **4a** Langmuir film (M = Cr) is shown in Figure 4. The **4a** complex exhibited at low surface pressures a molecular rearrangement with the formation of aggregates. Above 9.0 mN·m^−1^ the collapsed phase was observed, which agrees with the observation of a maximum in the isotherm, which indicates non-equilibrium processes.

**Figure 5:**
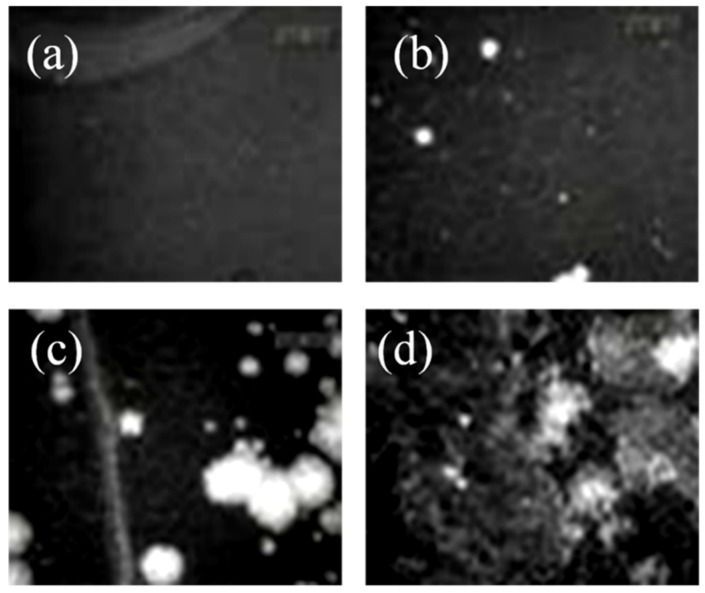
BAM images (width: 500 µm) of the **4a** compound at different surface pressures: (**a**) 0.5 mN·m^−1^, (**b**) 3.0 mN·m^−1^, (**c**) 7.0 mN·m^−1^, and (**d**) 14.0 mN·m^−1^.

The **4a** film was also characterized through BAM, Figure 5, where a progressive nucleation process can be observed [38,39]. Figure 5a–c showed that the surface of this film became rough as the compression increased, then an inhomogeneous and unstable monolayer appeared.

In the two-phase coexistence region (where a high-density condensed phase is mixed with a very low-density fluid phase) a variety of agglomerate sizes and shapes can be found producing a film with a poor homogeneity. These agglomerates on the surface depend not only on the chemical structure of the amphiphilic monolayer but also on the decomposition of the complex, which is stable only at low temperatures, between 2 and 4 °C. Finally, the collapse occurred due to the formation of this rough material, Figure 5d. The hysteresis plot could not be obtained since the agglomerates on the surface produced an unstable monolayer.

Results indicated that chromium-carbene compounds, **2a** and **4a**, produced isotherms which showed well-defined collapses at low surface pressures around 9.0 mN·m^−1^, with an irregular monolayer formation *i.e.*, low homogeneity and poor stability, because it was not possible to obtain their hysteresis. In the **2a** complex there is not reversibility suitable, which was attributed to the “non-return” of the organization molecular formed during compression to their original state after decompression, and for complex **4a**, any reversibility was obtained because several agglomerates were formed during the compression process [20].

#### 2.2.2. Langmuir Monolayers of Aminocarbene **2b** and **4b** Complexes, with Tungsten as Transition Metal

A second series of complexes with tungsten as transition metal were studied through the LB technique and then compared with those that contain chromium.

##### Langmuir Isotherm for the **2b** Complex

**Figure 6:**
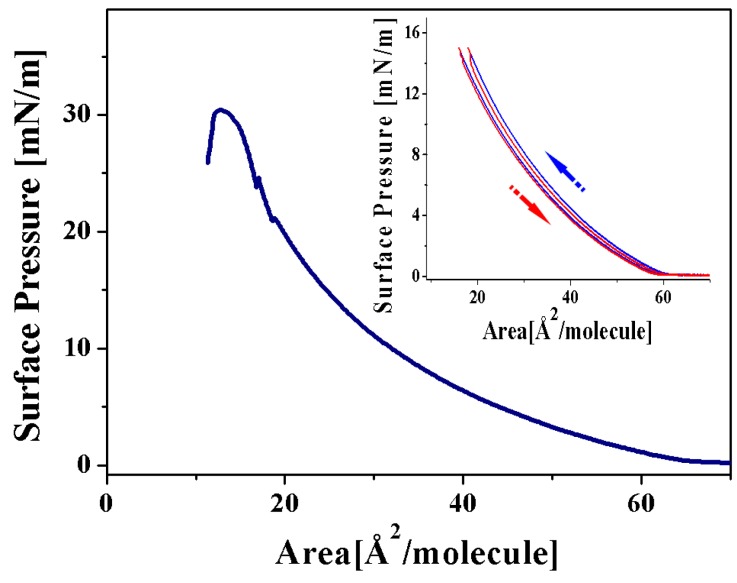
Surface pressure *vs.* molecular area isotherm of ferrocenyl aminocarbene complex **2b**, M = W. Inset shows reversibility of two successive compression-expansion cycles.

The isotherm of ferrocenyl aminocarbene **2b** complex, M = W confirmed the formation of a stable monolayer; no phase transition was observed (see Figure 6). In this figure, it can be observed that collapse begins at a surface pressure around 20.0 mN·m^−1^; for a surface pressure above 25.0 mN·m^−1^ the monolayer undergoes the formation of folds.

Hysteresis studies for **2b** L monolayer exhibited reversibility on successive compression/decompression cycles, the Figure 6 inset shows in the blue upward direction of compression and red the downward direction corresponding to the decompression. These results indicate the formation of a more stable monolayer for **2b** complex than the monolayer for **2a** complex, which was assigned to the organization of the molecules, they formed a monolayer during the compression process and the molecules follow the same path returning to their original state after decompression. However, the **2a** complex “non-return” to their original state after decompression.

**Figure 7:**
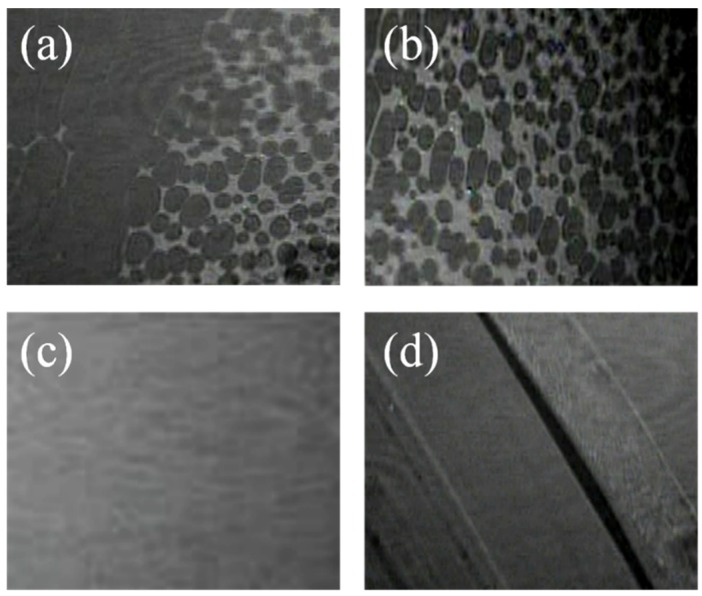
BAM images (width: 500 µm) of the **2b** compound at different surface pressures: (**a**) 0.5 mN·m^−1^, (**b**) 5.0 mN·m^−1^, (**c**) 10.0 mN·m^−1^, and (**d**) 25.0 mN·m^−1^.

BAM observations, Figure 7, confirmed the formation of **2b** ferrocene-derivative film. Indeed, when the surface pressure started to increase slowly large islands throughout the surface were formed Figure 7a,b. When the surface pressure was increased, it could be noticed that molecules begin to form a homogeneous monolayer in the air-water interface. Figure 7c, obtained at 10.0 mN·m^−1^, the film showed a uniform and homogeneous composition of the monolayer. Finally, at a higher surface pressure, approximately 25.0 mN·m^−1^, it was possible to observe a transition in which the 2D film goes to a more stable 3D phase [36,37]; therefore, the monolayer collapsed via a folding mechanism, Figure 7d.

##### Langmuir Isotherm for the **4b** Complex

Phenyl aminocarbene **4b** complex, M = W, was developed as a L film at the air-water interface. The surface pressure *versus* molecular area isotherm was recorded under similar conditions than those for **2**(**a-b**) films. BAM observations were also carried out for these films. 

**Figure 8:**
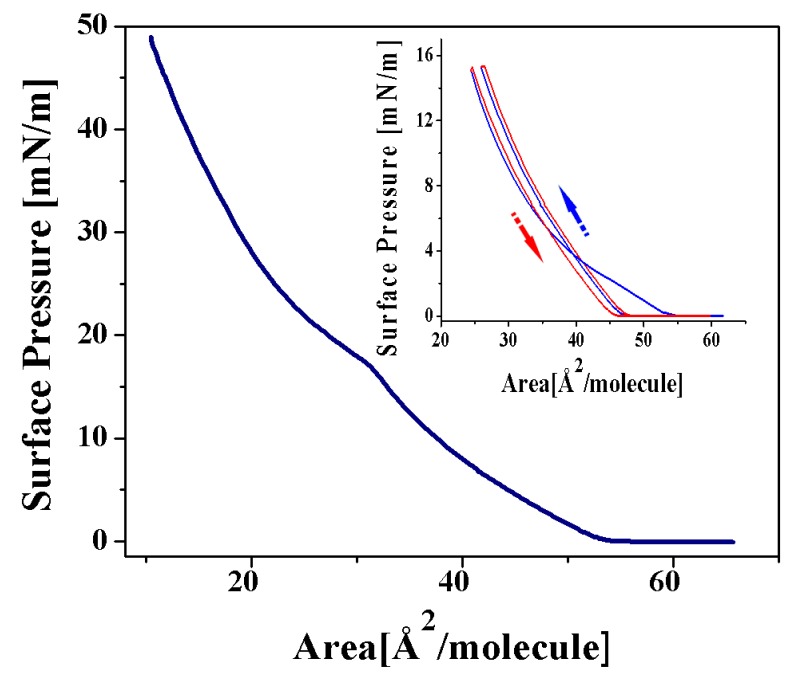
Surface pressure *vs.* molecular area isotherm of phenyl aminocarbene complex **4b**, M = W. Inset shows the reversibility of two successive compression-expansion cycles.

From the isotherm, Figure 8, a rearrangement of the molecules at the air-water interface for a surface pressure above 16.0 mN·m^−1^ was observed. The change in the slope at this value indicated that the collapse of the monolayer begun. Figure 8 inset, shows, in blue, upward direction of compression, and red, the downward direction corresponding to the decompression; the monolayer also exhibited reversibility on successive compression/decompression cycles.

**Figure 9:**
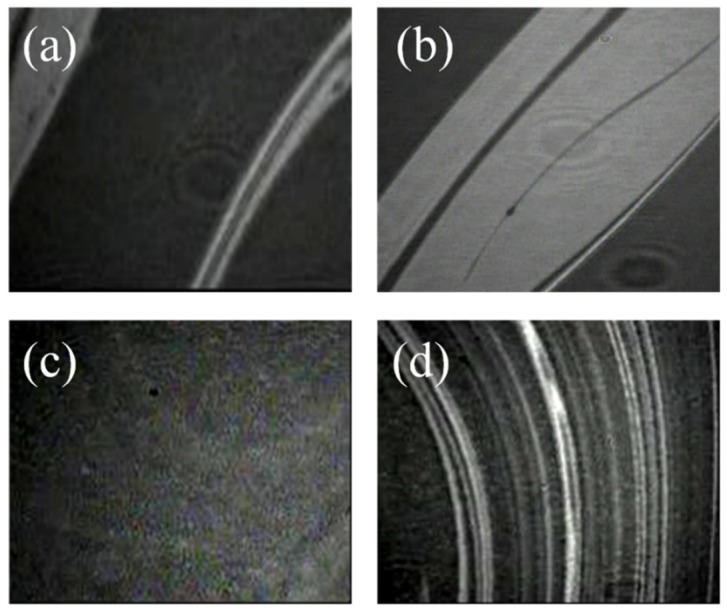
BAM images (width: 500 µm) of the **4b** compound at different surface pressures: (**a**) 0.5 mN·m^−1^, (**b**) 3.0 mN·m^−1^, (**c**) 12.0 mN·m^−1^, and (**d**) 35.0 mN·m^−1^.

The first two BAM micrographs, Figure 9a,b, show the growth of bands to form an L monolayer. Figure 9c, exhibits a monolayer with some spots or domains. When the compression increased, above 25.0 mN·m^−1^, the formation of some ridges was observed, Figure 9d.

Isotherms for the tungsten-carbene compounds, **2b** and **4b**, showed a better formation for both monolayers; it was found that the collapse begins at a surface pressure above 16.0 mN·m^−1^ a higher value than that for chromium carbene compounds (9.0 mN·m^−1^). For these two complexes the hysteresis plots showed a better stability of monolayers.

BAM results showed that the **4b** L monolayer, M = W, is more homogeneous than that for the **4a** complex, M = Cr.

### 2.3. Langmuir–Blodgett films

Langmuir films were transferred onto solid substrate through the LB method to form Z-type multilayers. Due to their poor homogeneity, **2a** and **4a** complexes did not form appropriate monolayers, and in this case they were not transferred as LB films. On the other hand, **2b** and **4b** complexes, both containing tungsten, which exhibited good homogeneity could be transferred onto solid substrates. 

**Figure 10:**
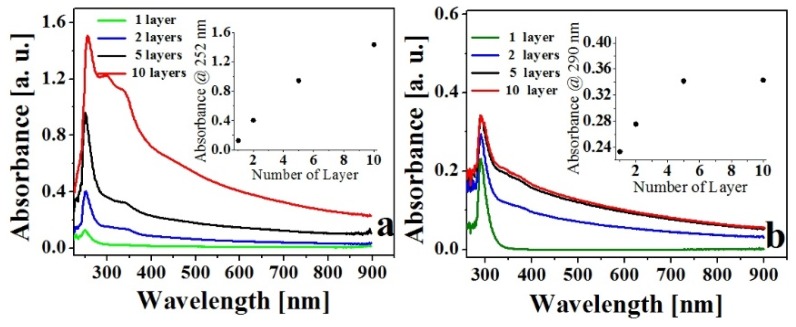
UV–VIS absorption spectra of LB films with 10 layers: (**a**) **2b** and (**b**) **4b**. Inset: Absorbance *versus* the number of layers at: (**a**) 252 nm and (**b**) 290 nm.

The characterization of **2b** and **4b** LB films were carried out by plotting their absorbance, Figure 10. Insets of Figure 10, show the absorbance as a function of the number of layers. In the first case, for sample **2b** at 252 nm, this figure shows a linear increase of absorption at this particular wavelength. This result indicates an efficient transfer of the monolayers onto the glass substrate, as well as the conservation of the molecular arrangement for this complex. However, for **4b** compound a stable molecular arrangement was observed only for the film with five layers; above six layers, the linear relationship was lost, Figure 10b.

These results showed that the molecular arrangement of the film which was obtained from the **2b** ferrocenyl hexadecylamino Fischer carbene complex showed higher stability and homogeneity than the **4b** the phenyl hexadecylamino Fischer carbene complex.

### 2.4 Atomic Force Microscopy (AFM)

**Figure 11:**
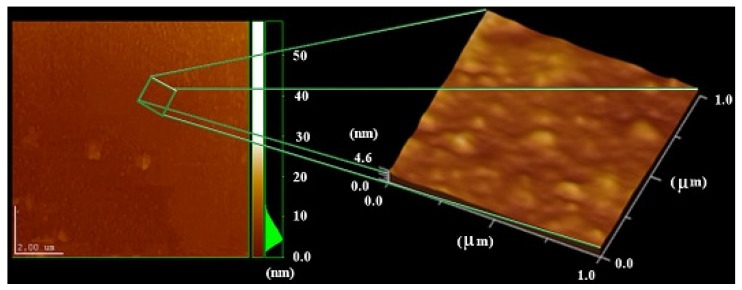
AFM micrograph of **2b** monolayer film**:** frontal and 3D views, respectively.

Variations in the morphology and roughness of **2b** and **4b** LB films, both containing tungsten, were studied by AFM. Results indicated that the **2b** monolayer complex is very homogeneous, in good agreement with the results obtained by Brewster angle microscopy during the *in situ* film formation. The high homogeneity film and low absolute roughness (*R_a_*) for **2b** film is observed in Figure 11, where *R_a_* = 2.9 nm.

**Figure 12:**
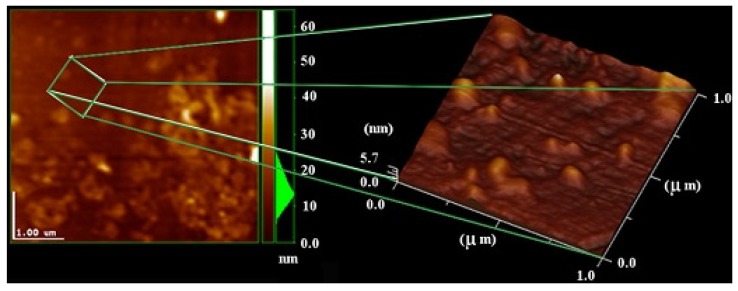
AFM micrographs of **4b** monolayer film: frontal and 3D views, respectively.

**Figure 13:**
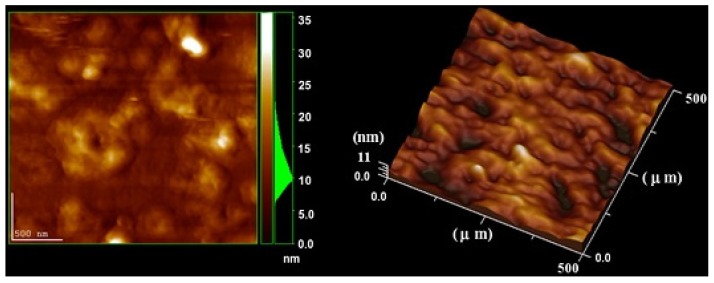
AFM micrographs of **4b** double-layer film: Frontal and 3D views, respectively.

For the **4b** complex, the homogeneity decreased, Figure 12. Small domains appeared, probably due to the interactions of the polar heads of this complex, in this case *R_a_* = 3.5 nm. As the number of monolayers increased (e.g., from mono to double-layer) more domains were observed on the surface. Figure 13 shows the formation of small 3D structures on the surface of these films due to the increased number of domains.

**Figure 14:**
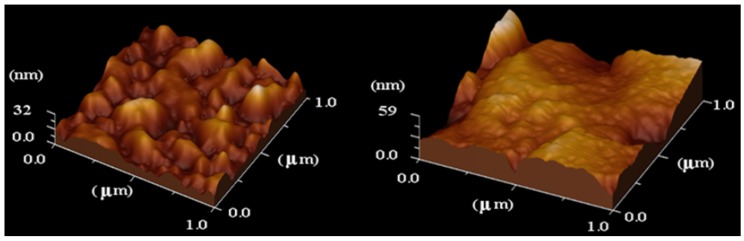
3D AFM micrographs with ten monolayers for **2b** and **4b** films, respectively.

For the ten layer LB film of **4b** complex, the molecular order disappeared due to the formation of a large number of domains and the formation of crests started. The presence of these domains could be the reason why the absorbance of **4b** sample, Figure 10b, had a nonlinear behavior. Finally, the morphology studied through AFM showed a well-ordered surface for all the **2b** LB films, including the film with ten layers, Figure 14.

### 2.5 Small-Angle X-ray Scattering (SAXS)

**Figure 15:**
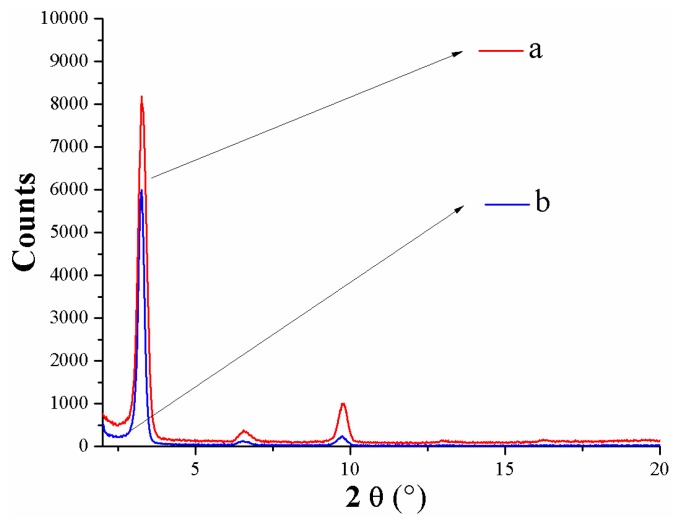
Small-angle X-ray scattering of a 10-layer Langmuir–Blodgett film of **2b** complex: a—four days and b—12 months, after deposition.

SAXS experiments were carried out for **2b** LB films, which were deposited onto glass plates. Diffractograms for the ten layers **2b** film, after four days and 12 months of deposition, are presented in Figure 15. Results show that this LB film tends to an equilibrium, which was reached only few days after deposition. These data proved the formation of highly ordered and stable LB films similar to those observed for ferrocenyl derivatives [40].

## 3. Experimental Section

### 3.1. Materials

Solvents and precursors used in this study were Aldrich products. Acetone was distilled over calcium chloride; tetrahydrofuran was distilled over sodium and benzophenone under a nitrogen atmosphere. Column chromatography was performed with Merck silica gel (70–230 mesh) and neutral alumina. To analyze their purity, all compounds were characterized by NMR spectroscopy. The ^1^H and ^13^C NMR spectra were recorded on a JEOL Eclipse 300 spectrometer (JEOL Ltd., Tokyo, Japan), using CDCl_3_ as a solvent and TMS as an internal reference. Chemical shifts are presented in ppm (δ). IR spectra were performed on a Perkin-Elmer 283 B or 1420 spectrometer (PerkinElmer Inc., Waltham, MA, USA). Melting points were obtained on Melt-Temp II equipment (Barnsted Thermolyne, Dubuque, IA, USA). The MS-FAB and MS-EI spectra were obtained with a JEOL SX 102A spectrometer (JEOL Ltd., Tokyo, Japan). The UV–VIS spectra were obtained on a CaryWin 100 Fast-Scan-Varian spectrophotometer (Varian, CA, USA), using fresh solutions of **2a** and **2b** in CHCl_3_ spectrophotometric grade. Molar concentrations were prepared in the ranges 8.76 × 10^−6^–4.38 × 10^−5^ mol/L for the **2b** complex and 8.81 × 10^−6^ mol/L–5.10 × 10^−5^ mol/L for the **4b** complex. 

### 3.2. Synthesis of Organometallic Precursors

#### 3.2.1. Preparation of Fischer Ferrocenyl Hexadecylamino **2**(**a-b**) Complexes

Fischer ethoxy ferrocenyl carbene **1**(**a-b**) complexes were prepared following a process described in a previous report [29]. Hexadecylamine (1.2 equiv, 2.76 mmol) was added to a solution of **1** (2.3 mmol) in anhydrous ether (10 mL) at room temperature, and then the reaction was stirred overnight. The solvent was evaporated under vacuum and the product was purified through chromatography on alumina using hexane as eluent to obtain an orange solid.

[(Ferrocenyl)(hexadecylamine)methylidene] pentacarbonyl chromium (0) **2a**: C_32_H_43_NO_5_CrFe, orange solid, mp: 80–84 °C, yield: 90%. ^1^H NMR (CDCl_3_) δ: 9.47 (s, 1H, NH), 4.44 (br s, 4H, Cp_subst_), 4.19 (s, 5H, Cp), 4.04 (m, 2H, NC*H*_2_), 1.86 (m, 2H, NCH_2_C*H*_2_), 1.26 (br s, 26H, C*H*_2_), 0.88 (br s, 3H, CH_3_). ^13^C NMR (CDCl_3_) δ: 270.4 (C=Cr), 223.6 (CO), 217.9 (CO), 99.4 (C_ipso_, Cp), 70.1 (CH, Cp_subst_), 69.4 (Cp), 68.4 (CH, Cp_subst_), 53.0 (N*C*H_2_), 32.0 (NCH_2_*C*H_2_), 30.0 (NCH_2_CH_2_*C*H_2_), 29.7 (10C, *C*H_2_), 27.0 (*C*H_2_CH_2_CH_3_), 22.8 (*C*H_2_CH_3_), 14.2 (*C*H_3_). IR (CHCl_3_) ν_max_ cm^−1^: 3306 (N–H), 2050 (CO), 1963 (CO), 1898 (CO), 1858 (CO). MS (FAB^+^) *m*/*z*: 573 [(M^+^− 2(CO)], 489 [M^+^ − 5(CO)].

[(Ferrocenyl)(hexadecylamine)methylidene] pentacarbonyl tungsten (0) **2b**: C_32_H_43_NO_5_WFe, orange solid, mp: 74–75 °C, yield: 88%. ^1^H NMR (CDCl_3_) δ: 9.0 (s, 1H, NH), 4.50 (m, 4H, C*H*_2_–N and C*H* Cp_subst_), 4.18 (br s, 5H, Cp), 3.89 (br s, 2H, CH Cp_subst_), 1.81 (m, 2H, NCH_2_C*H*_2_), 1.47–1.24 (m, 26H, C*H*_2_), 0.86 (br s, 3H, CH_3_). ^13^C NMR (CDCl_3_) δ: 249.9 (C=W), 203.2 (WCO), 198.7 (CO), 97.4 (C_ipso_, Cp), 70.9 (CH, Cp_subst_), 69.7 (Cp), 69.6 (CH, Cp_subst_) 55.3 (N*C*H_2_), 32.0 (NCH_2_*C*H_2_), 29.8 ((CH_2_)_11_), 27.0 (*C*H_2_CH_2_CH_3_), 22.8 (*C*H_2_CH_3_), 14.23 (CH_3_). IR (CHCl_3_) ν_max_ cm^−1^: 3312 (N–H), 2058 (CO), 1961 (CO), 1934 (CO), 1893 (CO), 1864 (CO), 1853 (CO). MS (FAB^+^) *m/z*: 761, 760 [M^+^ + 1], 705 [(M^+^) − 2(CO)], 677 [(M ^+^) − 3(CO)].

#### 3.2.2. Preparation of Fischer Hexadecylamino Phenyl Carbene **4**(**a-b**) Complexes

Fischer ethoxy phenyl carbene **3**(**a-b**) complexes were prepared following a process described in the literature [26]. Hexadecylamine (2.2 mmol) was added to a solution of **3** (2 mmol) in anhydrous ether (10 mL) at room temperature, and then the reaction was stirred overnight. The solvent was evaporated under vacuum and then the product was purified through chromatography on alumina using hexane as eluent to obtain a stable yellow compound.

[(Phenyl)(hexadecylamine)methylidene]pentacarbonyl chromium (0), **4a**: C_28_H_39_NO_5_Cr, yellow oil, yield: 90% (*E*/*Z*, 6:4). ^1^H NMR (CDCl_3_) δ: 9.0 (s, 1H, NH, isomer *E*), 8.55 (s, 1H, NH, isomer *Z*), 8.0 (m, 1H, CH_arom_), 7.58–7.16 (m, CH_arom_) 6.95 (m, 1H, CH_meta_ isomer *Z*), 6.78 (m, 1H, CH_meta_ isomer *E*), 4.03 (m, 2H, CH_2_–N, isomer *Z*), 3.16 (m, 2H, CH_2_–N, isomer *E*), 1.79 (m, 2H, CH_2_CH_2_N), 1.26 (m, 26 H, –CH_2_–), 0.88 (t, 3H, CH_3_). ^13^C NMR (CDCL_3_) δ: 281.0 (C=Cr, isomer *E*), 277.7 (C=Cr, isomer *Z*), 223.8, 223.4 [Cr(CO)], 217.3 (Cr–CO), 155.1 [C_ipso_ (isomer *Z*)], 149.6 [C_ipso_ (isomer *E*)], 128.5, 126.6 and 119.0 [CH_arom_ (isomer *E*)], 128.7, 128.2 and 120.9 [CH_arom_ (isomer *Z*)], 53.6 [CH_2_–N, (isomer *Z*)], 50.9 [CH_2_–N, (isomer *E*)], 31.9, 29.6, 29.2,26.6,26.2 and 22.7 (–CH_2_–), 14.1 (CH_3_). IR (CHCl_3_) ν_max_ cm^−1^: 3355 and 3290 (N–H), 2054, 1974, 1889 (MCO). MS (FAB^+^) *m/z*: 493 [(M^+^) − (CO)], 381 [(M^+^) − 5(CO)].

[(Phenyl)(hexadecylamine)methylidene] pentacarbonyl tungsten (0) **4b**: C_28_H_39_NO_5_W, yellow solid, mp: 43–44 °C, yield: 85% (*E*/*Z*, 8:1). ^1^H NMR (CDCl_3_) δ: 9.01 (s, 1H, NH, isomer *E*), 8.62 (s, 1H, NH, isomer *Z*), 7.72 (m, 1H, CH_arom_), 7.58–7.16 (m, 5H, CH_arom_); 7.06–7.02 (m, 1H, CH_meta_ isomer *Z*), 6.8–6.78 (m, 1H, CH_meta_ isomer *E*), 4.04 (q, 2H, CH_2_–N, isomer *Z*), 3.43 (q, 2H, C*H*_2_–N, isomer *E*), 1.85 (m, 2H, C*H*_2_CH_2_N); 1.29 (br m, 22H, –CH_2_–) 0.88 (t, 3H, CH_3_). ^13^C NMR (CDCl_3_) δ: 258.0 (C=W), 203.8 (W–CO), 199.1 (W–CO), 155.4 (C_ipso_), 131.4, 128.5, 128.2, 126.8, 120.8 (CH_arom_), 55.8 (*C*H_2_–N), 31.9–22.7 (14–CH_2_–), 14.2 (CH_3_). IR (CHCl_3_) ν_max_ cm^−1^: 3344 and 3289 (N–H), 2062, 1972, 1895 and 1880 (M–CO). MS (FAB^+^) *m/z*: 653[(M^+^)], 625[M^+^ − (CO)], 597 [(M ^+^) − 2(CO)].

### 3.3. Preparation of Langmuir Films

The spreading solution was prepared in chloroform with a specific concentration of 1 mg·mL^−1^. ASTM type 1 ultra-pure water (Millipore-Q system, 18.2 MΩcm) was used for the subphase. Studies on the monolayer were carried out with a KSV 5000 trough system 3 (KSV, Helsinki, Finland), slowly spreading suitable amounts of solution on the water surface with a microsyringe (100 µL). After spreading, the monolayer was maintained for 10 min at room conditions for solvent evaporation. Afterwards, it was symmetrically compressed with a barrier speed of 5 mm·min^−1^. The surface pressure measurement, which was performed according to the Wilhelmy method [20,21,22], and the isotherm were recorded at 25 °C. Finally, the stability of monolayers was studied through repetitive compression-expansion processes (hysteresis loops) without exceeding the collapse pressure.

#### Characterization of Langmuir Films through BAM

The quality of the Langmuir films was monitored using a Mini BAM-Plus system from Nanofilm Technology GmbH (Goettingen, Germany). This system is equipped with a 660 nm laser source working at 30 mW. The film quality observations were carried out at the Brewster angle (around 53.15° for an air-water interface) incidence [41,42]. During these experiments, high-resolution images were directly acquired with a built-in CCD camera.

### 3.4. Preparation of LB Films

The Langmuir monolayers obtained from ferrocene derivatives were transferred onto solid substrates using the Langmuir–Blodgett technique with a vertical lifting method. The transfer ratio value obtained was 1.0 ± 0.1. Films were deposited at 25 °C, using 75 × 75 × 1 mm^3^ Pyrex glass plates as substrates. All glass slides were successively treated with a sulfocromic mixture solution, ultrapure water, ethanol (reagent grade, Aldrich, St. Louis, MO, USA) and, finally, chloroform (reagent grade, Aldrich, St Louis, MO, USA). They were subsequently stored under clean and dry conditions until film deposition. Finally, *Z*-type multilayered structures with *n* = 1, 2, 5, and 10 layers were prepared experimentally [21,22] through extraction at a target pressure in the range of 10−12 mN·m^−1^ and a dipping speed of 5 mm·min^−1^, waiting 10 min between successive dipping cycles in order to evaporate the trapped subphase.

#### Characterization of LB Films

UV-VIS spectra of glass slides with LB films were obtained with a double beam Varian Cary 100 Fast-Scan spectrophotometer (Varian, CA, USA), using a glass slide without LB films as a reference. Unfortunately, the conductive measures of the LB films obtained in this work cannot be obtained because of the limitations of the technique with regard with the thickness of LB films obtained. The diffractograms were obtained with a XRD grazing incidence studies (1°) were carried out with a Rigaku ULTIMA-IV diffractometer Rigaku, Tokio, Japan, 40 kV, 44 mA with Cu*K*α radiation and AFM images of LB films deposited on glass substrates were acquired using a JEOL-JSPM-4210 atomic force microscope (JEOL Ltd, Akishima-Tokyo, Japan), in tapping mode with a NSC12 rectangular cantilever (µmash ^TM^) and a resonance frequency of 318 kHz (NanoSensors). Scans were performed at room temperature and atmospheric pressure.

## 4. Conclusions 

The synthesis of two series of push-pull organometallic molecules using a Fischer aminocarbene complex RC = [M(CO)_5_]NH(CH_2_)_15_CH_3_, [**2(a-b)**: with R = Fc and M = Cr, W] and [**4(a-b)**: with R = Ph and M = Cr, W] were prepared. For the first group, films of **2b** complex from one to ten layers were deposited, and for the second group, films of **4b** complex from one to five layers were obtained. The best results in all analyses were found for samples **2b** and **4b**, since **2a** and **4a** films were irregular and they formed defects and agglomerates during the Langmuir film formation. For Langmuir films obtained from **2b** and **4b** complexes, results from BAM showed that the Langmuir monolayer of **2b** is very homogeneous in the condensed phase. This behavior could correlate with the spectroscopic evidence of the **2b**, which indicate that this amino carbene complex exists solely as a Z-isomer, and this conformation promotes a better molecular arrangement in the Langmuir film.

The UV-VIS spectrum for the 10 layer LB films of **2b** showed a better molecular arrangement in comparison to the five layer LB films of **4b**. These results also indicated that the monolayers of ferrocenyl aminocarbene complexes (**2a**, **2b**) exhibited more stability than the monolayers of phenyl aminocarbene complexes (**4a**, **4b**).

AFM results for the **2b** LB monolayer films showed a homogeneous surface. However, the **4b** LB films had a surface with nanodomains and several large solid domains, which produced the loss of structural arrangement as the number of deposited monolayers increased.

AFM data confirmed the results obtained through BAM, since both techniques provided evidence about the molecular arrangement of the films. Results showed that **2b** ferrocenyl aminocarbene films exhibited the best stability and homogeneity of all samples, probably due to the larger stability in the electronic interaction between the conjugate bonds of ferrocenyl and the π-bonded carbene carbon-tungsten atoms, which make these nanomaterials suitable for potential electronic applications.
